# New Azalomycin F Analogs from Mangrove *Streptomyces* sp. 211726 with Activity against Microbes and Cancer Cells

**DOI:** 10.3390/md11030817

**Published:** 2013-03-12

**Authors:** Ganjun Yuan, Kui Hong, Haipeng Lin, Zhigang She, Jia Li

**Affiliations:** 1 College of Bioscience and Engineering, Jiangxi Agricultural University, 1101 Zhimin Road, Nanchang 330045, China; E-Mail: sqlygj@126.com; 2 Institute of Tropical Bioscience and Biotechnology, Chinese Academy of Tropical Agriculture Sciences, Haikou 571101, China; E-Mail: linhp010612@gmail.com; 3 Key Laboratory of Combinatorial Biosynthesis and Drug Discovery (Ministry of Education), School of Pharmaceutical Sciences, Wuhan University, Wuhan 430071, China; 4 School of Chemistry and Chemical Engineering, Sun Yat-Sen University, Guangzhou 510275, China; E-Mail: cesshzhg@mail.sysu.edu.cn; 5 National Center for Drug Screening, Shanghai Institute of Materia Medica, Shanghai 201203, China; E-Mail: jli@mail.shcnc.ac.cn

**Keywords:** azalomycin F, *Streptomyces* sp. 211726, cytotoxicity, antimicrobial activity

## Abstract

Seven new azalomycin F analogs (**1**–**7**) were isolated from the broth of mangrove *Streptomyces* sp. 211726, and respectively identified as 25-malonyl demalonylazalomycin F_5a_ monoester (**1**), 23-valine demalonylazalomycin F_5a_ ester (**2**), 23-(6-methyl)heptanoic acid demalonylazalomycins F_3a_ ester (**3**), F_4a_ ester (**4**) and F_5a_ ester (**5**), 23-(9-methyl)decanoic acid demalonylazalomycin F_4a_ ester (**6**) and 23-(10-methyl)undecanoic acid demalonylazalomycin F_4a_ ester (**7**). Their structures were established by their spectroscopic data and by comparing with those of azalomycins F_3a_, F_4a_ and F_5a_. Biological assays exhibited that **1**–**7** showed broad-spectrum antimicrobial and anti HCT-116 activities.

## 1. Introduction

**Figure 1:**
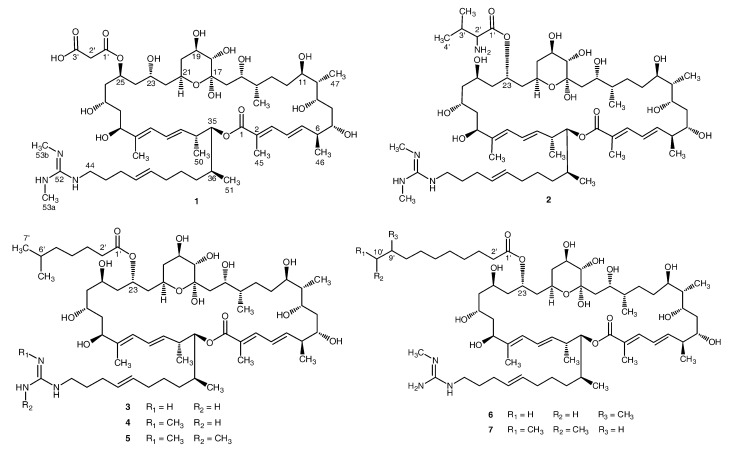
Structure of compounds **1**–**7** from Mangrove *Streptomyces* sp. 211726.

Mangroves are woody plants located in tropical and subtropical intertidal coastal regions, which are high productive ecosystems [1,2]. Novel bioactive compounds have been reported from the plant materials [3,4,5]. Mangrove streptomycetes are also potential resources for the discovery of anti-infection, anti-tumor and hypoglycemic compounds [6,7,8,9,10]. *Streptomyces* sp. 211726, a remarkable producer of macrocyclic lactones, was selected from 288 strains when we carried on the chemical screening for macrolide-producing mangrove actinomycetes. Five azalomycin F analogs including azalomycins F_3a_, F_4a_, F_5a_, azalomycin F_4a_ 2-ethylpentyl ester and azalomycin F_5a_ 2-ethylpentyl ester were identified from the culture broth of this strain in our previous work [11], while the HPLC profiles of the methanol extract and several macrolide constituents indicated that many azalomycin F analogs were produced by this strain. After the relative configurations of azalomycins F_3a_, F_4a_ and F_5a_ were assigned [12], further research on minor azalomycin F analogs produced by this strain led to seven new compounds (Figure 1) which were respectively identified as 25-malonyl demalonylazalomycin F_5a_ monoester (**1**), 23-valine demalonylazalomycin F_5a_ ester (**2**), 23-(6-methyl)heptanoic acid demalonylazalomycins F_3a_ (**3**), F_4a_ (**4**) and F_5a_ (**5**) esters, 23-(9-methyl)decanoic acid demalonylazalomycin F_4a_ ester (**6**) and 23-(10-methyl)undecanoic acid demalonylazalomycin F_4a_ ester (**7**). Their structures were established by their spectroscopic data (IR, UV, NMR, MS) and by comparing with those of azalomycins F_3a_, F_4a_ and F_5a_ which were reported in our previous paper [11], and their complete ^1^H and ^13^C assignments were achieved by using ^1^H, ^13^C, DEPT, HSQC, ^1^H-^1^H COSY and HMBC spectra in MeOH-*d*_4_. Moreover, biological assays of **1**–**7** showed broad-spectrum antimicrobial activity as well as anti HCT-116 activity.

## 2. Results and Discussion

### 2.1. Structural Elucidation

25-Malonyl demalonylazalomycin F_5a_ monoester (**1**) was obtained as a white, amorphous powder with [α]_D_^29^ +6.7° (*c* 0.1, MeOH). Its molecular formula C_57_H_97_N_3_O_17_ was established by the HRESIMS spectrometric data at *m*/*z* 1096.6914 [M + H]^+^ (calcd. for C_57_H_98_N_3_O_17_, 1096.6896), which showed that its molecular formula was identical to that of azalomycin F_5a_. Like azalomycin F_5a_, its UV absorption maxima at 241 nm (log ε, 4.6) and 269 nm (log ε, 4.3) also indicated the presence of a conjugated diene and an α,β,γ,δ-unsaturated acid (or ester) group. The ^13^C, DEPT and HSQC spectra of **1** (Table 1) showed one guanidino carbon signal at δ_C_ 157.42, three carbonyl carbon signals at δ_C_ 170.2, 171.9 and 173.9, ten olefinic carbon signals at δ_C_ 125.3, 126.8, 127.6, 128.6, 130.3, 132.6, 136.3, 140.2, 140.3 and 146.2, one quaternary hemiacetical carbon at δ_C_ 99.9 and one methine carbon at δ_C_ 70.8. So, **1** was deduced as an isomer of azalomycin F_5a_. When we compared the ^13^C and DEPT spectra of **1** with those of azalomycin F_5a_ [13], the signal at δ_C_ 46.6 (C-26) in the ^13^C NMR spectrum of azalomycin F_5a,_ was not observed while a signal at δ_C_ 44.0 appeared in that of **1**. Based on the HSQC, ^1^H-^1^H COSY and HMBC spectra of **1**, the linking position of the malonyl group was assigned to C-25 in **1**, and the signal at δ_C_ 44.0 was assigned to C-26. It is interesting that **1** was found to be convertible to azalomycin F_5a_. HPLC analysis showed that the ratio of **1** to azalomycin F_5a_ was about 15:85 after **1** stood in MeOH-*d*_4_ at room temperature for 30 days. This phenomenon was also observed by Iwasaki S. *et al.* [14]. The compound convertible to azalomycin F_5a_ was named as azalomycin F_5b_, although spectroscopic information and structure of azalomycin F_5b_ was not given in the paper [14]. There is not enough evidence to confirm that **1** and azalomycin F_5b_are the same compound. So, **1** was identified as 25-malonyl demalonylazalomycin F_5a_ monoester.

**Table 1:** NMR spectroscopic data (400 MHz for ^1^H, 100 MHz for ^13^C) of **1**, **2**, **3** and **6** in MeOH-*d*_4_ (δ in ppm).

Position	1			2		3			6		
	δ_C_		δ_H_ (*J* in Hz)	δ_C_	δ_H_ (*J* in Hz)	δ_C_	δ_H_ (*J* in Hz)		δ_C_	δ_H_ (*J* in Hz)	
C-1	170.2		-	170.1	-	170.1	-		170.1	-	
C-2	126.8		-	126.7	-	126.8	-		126.8	-	
C-3	140.3		7.09 d (11.2)	140.3	7.10 d (11.0)	140.2	7.10 d (11.2)		140.3	7.10 d (11.0)	
C-4	127.6		6.43 dd (11.5, 14.9)	127.6	6.43 dd (11.5, 14.7)	127.6	6.43 dd (11.9, 14.3)		127.6	6.43 dd (11.5, 14.8)	
C-5	146.2		6.07 dd (15.1, 9.0)	146.1	6.08 dd (14.8, 9.0)	146.1	6.08 dd (14.0, 9.0)		146.1	6.08 dd (14.9, 9.0)	
C-6	44.8		2.43 m	44.7	2.44 m	44.6	2.43 m		44.7	2.44 m	
C-7	75.9		3.80 m	75.8	3.77 m	75.8	3.77 m		75.8	3.77 m	
C-8	39.3		1.50 m, 1.78 m	39.3	1.50 m, 1.78 m	39.3	1.50 m, 1.77 m		39.3	1.50 m, 1.78 m	
C-9	75.4		3.80 m	75.2	3.80 m	75.2	3.80 m		75.2	3.80 m	
C-10	44.7		1.54 m	44.6	1.53 m	44.5	1.54 m		44.6	1.53 m	
C-11	72.2		3.91 m	72.2	3.87 m	72.2	3.91 m		72.2	3.87 m	
C-12	33.5		1.62 m, 1.38 m	33.5	1.60 m, 1.38 m	33.5	1.62 m, 1.37 m		33.4	1.62 m, 1.37 m	
C-13	30.7		1.30 m, 1.45 m	30.6	1.30 m, 1.42 m	30.6	1.30 m, 1.43 m		30.6	1.30 m, 1.43 m	
C-14	40.6		1.60 m	40.5	1.61 m	40.7	1.61 m		40.5	1.61 m	
C-15	72.7		3.86 m	72.7	3.87 m	72.4	3.86 m		72.7	3.87 m	
C-16	41.9		1.80 m	41.8	1.81 m	41.9	1.82 m		41.8	1.81 m	
C-17	99.9		-	100.0	-	99.8	-		99.9	-	
C-18		77.5	3.34 d (9.2)	77.4	3.35 d (9.1)	77.2		3.35 d (9.2)	77.5	3.35 d (9.1)	
C-19		69.9	3.87 m	69.9	3.88 m	69.7		3.87 m	69.8	3.88 m	
C-20		41.4	1.89 m, 1.30 m	41.3	1.89 m, 1.30 m	41.2		1.90 m, 1.31 m	41.3	1.89 m, 1.31 m	
C-21		65.7	4.17 m	66.2	4.16 m	66.3		4.16 m	66.3	4.16 m	
C-22		44.5	1.52 m	41.9	1.82 m	41.8		1.88 m	41.9	1.81 m	
C-23		66.3	4.03 m	70.9	5.29 m	70.7		5.27 m	70.9	5.29 m	
C-24		44.6	1.69 m	44.0	1.70 m	44.0		1.72 m	44.1	1.76 m, 1.66 m	
C-25		70.8	5.28 m	65.7	3.86 m	65.6		3.87 m	65.7	3.86 m	
C-26		44.0	1.61 m, 1.83 m	46.3	1.51 m	46.4		1.51 m	46.3	1.51 m	
C-27		65.7	3.88 m	65.8	4.04 m	65.6		4.04 m	65.8	4.04 m	
C-28		44.2	1.78 m	44.1	1.54 m	44.1		1.53 m	44.1	1.63 m	
C-29		74.2	4.18 m	74.2	4.18 m	74.2		4.18 m	74.2	4.18 m	
C-30		140.2	-	140.1	-	140.1		-	140.1	-	
C-31		125.3	5.98 d (10.4)	125.3	5.98 d (10.7)	125.2		5.98 d (10.5)	125.3	5.98 d (10.7)	
C-32		128.6	6.22 dd (10.9, 14.5)	128.5	6.23 dd (10.9, 14.9)	128.6		6.22 dd (10.9, 14.9)	128.5	6.23 dd (10.9, 14.8)	
C-33		136.3	5.43 m	136.3	5.44 m	136.2		5.45 m	136.3	5.44 m	
C-34		41.0	2.57 m	40.7	2.57 m	41.0		2.57 m	40.9	2.57 m	
C-35		80.9	4.78 dd (7.6, 4.0)	80.9	4.79 dd (7.7, 4.1)	80.8		4.78 dd (7.8, 3.9)	80.9	4.79 dd (7.6, 4.1)	
C-36		35.3	1.82 m	35.3	1.82 m	35.2		1.81 m	35.3	1.82 m	
C-37		34.4	1.15 m, 1.35 m	34.4	1.15 m, 1.35 m	34.5		1.15 m, 1.35 m	34.4	1.15 m, 1.35 m	
C-38		27.9	1.42 m	27.8	1.42 m	27.9		1.41 m	27.9	1.42 m	
C-39		33.6	1.99 m	33.6	1.99 m	33.6		1.99 m	33.6	1.99 m	
C-40		132.6	5.44 m	132.5	5.44 m	132.6		5.44 m	132.6	5.44 m	
C-41		130.3	5.44 m	130.3	5.44 m	130.1		5.50 m	130.2	5.44 m	
C-42		30.7	2.07 m	30.4	2.07 m	30.8		2.07 m	30.6	2.07 m	
C-43		29.9	1.67 m	29.8	1.64 m	29.8		1.64 m	29.8	1.64 m	
C-44		42.2	3.17 t (7.3)	42.0	3.15 t (7.1)	42.0		3.15 t (7.0)	42.0	3.15 t (7.1)	
C-45		12.9	1.92 s	12.9	1.92 s	12.9		1.92 s	12.9	1.92 s	
C-46		17.1	1.11 d (6.8)	17.1	1.12 d (6.8)	17.1		1.11 d (6.8)	17.1	1.12 d (6.8)	
C-47		10.5	0.89 d (6.9)	10.5	0.89 d (6.9)	10.5		0.89 d (6.9)	10.5	0.89 d (6.9)	
C-48		15.2	0.91 d (6.7)	15.3	0.92 d (6.7)	15.3		0.91 d (6.7)	15.3	0.92 d (6.7)	
C-49		13.1	1.65 s	13.1	1.65 s	13.3		1.65 s	13.1	1.65 s	
C-50		17.8	1.01 d (6.7)	17.9	1.00 d (6.7)	17.9		1.00 d (6.6)	17.9	1.00 d (6.8)	
C-51		14.4	0.94 d (6.7)	14.5	0.95 d (6.7)	14.4		0.94 d (6.7)	14.5	0.94 d (6.7)	
C-52		157.4	-	157.4	-	158.7		-	158.3	-	
C-53a		28.4	2.85 s	28.4	2.85 s				28.4	2.84 s	
C-53b		28.4	2.85 s	28.4	2.85 s						
C-1′		171.9	-	174.1	-	175.5		-	175.4	-	
C-2′		46.0	3.22 m	61.7	3.44 d (4.0)	35.0		2.36 t (7.4)	35.0	2.36 t (7.5)	
C-3′		173.9	-	30.8	2.28 m	26.0		1.62 m	26.0	1.61 m	
3′-CH_3_				17.9	1.02 d (6.8)						
C-4′				19.2	1.07 d (6.8)	30.4		1.42 m	30.3	1.35 m	
C-5′						40.3		1.18 m	30.4	1.31 m	
C-6′						29.2		1.29 m	30.8	1.30 m	
6′-CH_3_						23.8		0.88 d (6.6)			
C-7′						23.7		0.88 d (6.6)	28.5	1.29 m	
C-8′									40.3	1.17 m	
C-9′									29.2	1.31 m	
9′-CH_3_									23.1	0.89 d (6.8)	
C-10′									23.1	0.89 d (6.8)	

**Figure 2:**
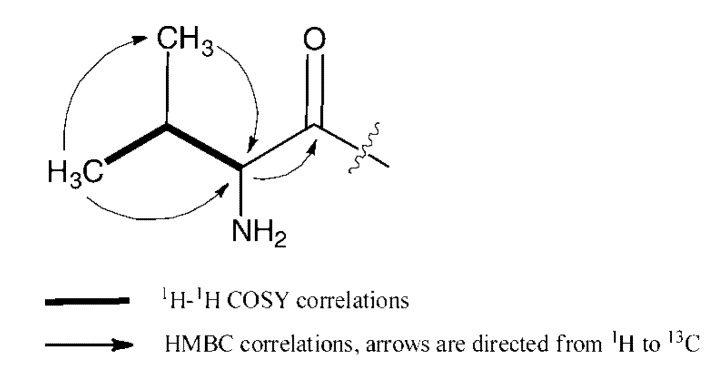
Key ^1^H-^1^H COSY and HMBC correlations of the valyl moiety in **2**.

23-Valine demalonylazalomycin F_5a_ ester (**2**) was obtained as a white, amorphous powder with [α]_D_^29^ +4.4° (*c* 0.1, MeOH). Its molecular formula C_59_H_104_N_4_O_15_ was established by the HRESIMS spectrometric data at *m*/*z* 1109.7580 [M + H]^+^ (calcd. for C_59_H_105_N_4_O_15_, 1109.7576). Its UV absorption maxima at 241 nm (log ε, 4.6) and 268 nm (log ε, 4.4) indicated the presence of a conjugated diene and an α,β,γ,δ-unsaturated acid (or ester) group. The ^13^C, DEPT and HSQC spectra of **2** (Table 1) showed one guanidino carbon signal at δ_C_ 157.4, one carbonyl carbon signal at δ_C_ 170.1, ten olefinic carbon signals at δ_C_ 125.3, 126.7, 127.6, 128.5, 130.3, 132.5, 136.3, 140.1, 140.3 and 146.1, one quaternary hemiacetical carbon signal at δ_C_ 100.0, one methine carbon signal at δ_C_ 70.9 and two *N*-methyl carbon signals at δ_C_ 28.4. These spectroscopic data were very similar to azalomycin F_5a_ reported in our previous paper [11], while there was no carbonyl carbon signal at δ_C_ 171.6 and methylene carbon signal at δ_C_ 46.1. Comparing the ^13^C, DEPT and HSQC spectra of **2** and those of azalomycin F_5a_, two additional methyl carbon signals at δ_C_ 17.9 and 19.2 and two methylene carbon signals at δ_C_ 30.81 and 61.7 were present. Based on the correlations (Figure 2) observed in the ^1^H-^1^H COSY and HMBC spectra of **2**, a valyl group was established. Moreover, the correlation between H-23 (δ_H_ 5.29) and C-1′ (δ_C_ 174.1) observed in the HMBC spectrum of **2** indicated that the valyl group was linked to the lactonic ring at C-23 with an ester bond. So, **2** was identified as 23-valine demalonylazalomycin F_5a_ ester.

**Figure 3:**
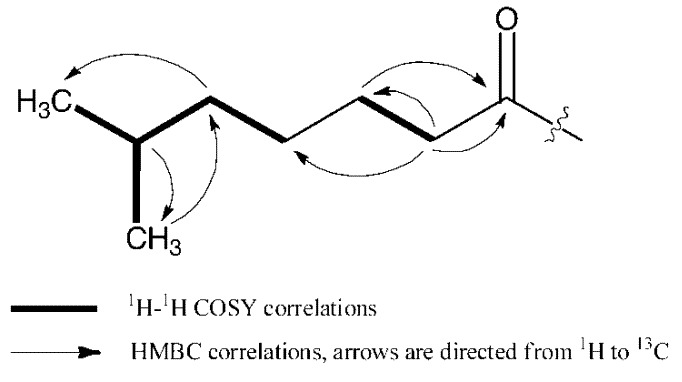
Key ^1^H-^1^H COSY and HMBC correlations of the 6-methyl heptanoyl moiety in **3**.

23-(6-Methyl)heptanoic acid demalonylazalomycin F_3a_ ester (**3**) was obtained as a white, amorphous powder with [α]_D_^20^ +6.8° (*c* 0.1, MeOH). Its molecular formula C_60_H_105_N_3_O_15_ was established by the HRESIMS spectrometric data at *m*/*z* 1108.7638 [M + H]^+^ (calcd. for C_60_H_106_N_3_O_15_, 1108.7624). Its UV absorption maxima at 238 nm (log ε, 4.6) and 269 nm (log ε, 4.3) indicated the presence of a conjugated diene and an α,β,γ,δ-unsaturated acid (or ester) group. The ^13^C, DEPT and HSQC spectra of **3** (Table 1) showed one guanidino carbon signal at δ_C_ 158.7, one carbonyl carbon signal at δ_C_ 170.1, ten olefinic carbon signals at δ_C_ 125.2, 126.8, 127.6, 128.6, 130.1, 132.6, 136.2, 140.1, 140.2 and 146.1, one quaternary hemiacetical carbon signal at δ_C_ 99.8 and one methine carbon signal at δ_C_ 70.7. These spectroscopic data were very similar to those of azalomycin F_3a_ [15], which were reported as supporting information in our previous paper [11], while there were no carbonyl carbon signals at δ_C_ 171.8 and 174.0 and methylene carbon signal at δ_C_ 45.8 in the ^13^C NMR spectrum of **3**. Comparing the ^1^H, ^13^C, DEPT and HSQC spectra of **3** with those of azalomycin F_3a_, two additional methyl carbon signals at δ_C_ 23.8 and 23.7, four methylene carbon signals at δ_C_ 40.3, 35.0, 30.4 and 26.0, one methine carbon signal at δ_C_ 29.2 and a carbonyl carbon signal at δ_C_ 175.5 were observed in the ^13^C NMR spectrum of **3**. Based on the correlations observed in the ^1^H-^1^H COSY and HMBC spectra (Figure 3), a 6-methyl heptanoyl group was deduced. Moreover, the correlations between H-23 (δ_H_ 5.27) and C-1′ (δ_C_ 175.5) observed in the HMBC spectrum of **3** indicated that the 6-methyl heptanoyl group was linked to the lactonic ring at C-23 with an ester bond. So, **3** was identified as 23-(6-methyl)heptanoic acid demalonylazalomycin F_3a_ ester.

23-(6-Methyl)heptanoic acid demalonylazalomycin F_4a_ ester (**4**) was obtained as a white, amorphous powder with [α]_D_^20^ +6.4° (*c* 0.1, MeOH). Its molecular formula C_61_H_107_N_3_O_15_ was established by the HRESIMS spectrometric data at *m*/*z* 1122.7788 [M + H]^+^ (calcd. for C_61_H_108_N_3_O_15_, 1122.7780). The difference of 14 mass units between **4** and **3** indicated that **4**has one methylene unit more than **3**. Similar ^1^H, ^13^C, DEPT spectra and UV absorption data allowed identification of these two compounds as analogs. Comparing the ^13^C and DEPT spectra of **4** with those of azalomycin F_4a_ [16], the guanidino carbon signal at δ_C_ 158.3 (C-52) indicated that one methyl group was linked to a guanidino nitrogen [11], which was also corroborated by a proton signal at δ_H_ 2.84 (3H, s, H-53a), a carbon signal at δ_C_ 28.4 (C-53a) and the correlation between H-53a and C-52 observed in the HMBC spectrum of **4**. So, **4** was identified as 23-(6-methyl)heptanoic acid demalonylazalomycin F_4a_ ester.

23-(6-Methyl)heptanoic acid demalonylazalomycin F_5a_ ester (**5)** was obtained as a white, amorphous powder with [α]_D_^20^ +6.1° (*c* 0.1, MeOH). Its molecular formula C_62_H_109_N_3_O_15_ was established by the HRESIMS spectrometric data at *m*/*z* 1136.7956 [M + H]^+^ (calcd. for C_62_H_110_N_3_O_15_, 1136.7937). The difference of 14 mass units between **5** and **4** indicated that **5** has one methylene unit more than **4**. Similar ^1^H, ^13^C, DEPT spectra and UV absorption data allowed identification of these two compounds as analogs. Comparing their ^1^H, ^13^C and DEPT spectra, the guanidino carbon signal at δ_C_ 157.4 indicated two methyl groups were linked to two guanidino nitrogens, which was also corroborated by proton signals at δ_H_ 2.84 (6H, s, H-53a and H-53b) and carbon signals at δ_C_ 28.4 (C-53a and C-53b) in the ^1^H and ^13^C NMR spectrum of **5**, respectively. So, **5** was identified as 23-(6-methyl)heptanoic acid demalonylazalomycin F_5a_ ester.

23-(9-Methyl)decanoic acid demalonylazalomycin F_4a_ ester (**6**) was obtained as a white, amorphous powder with [α]_D_^20^ +6.0° (*c* 0.1, MeOH). Its molecular formula C_64_H_113_N_3_O_15_ was established by the HRESIMS spectrometric data at *m*/*z* 1164.8269 [M + H]^+^ (calcd. for C_64_H_114_N_3_O_15_, 1164.8250). The difference of 42 mass units between **6** and **4** indicated that **6** has three methylene units more than **4**. Similar ^1^H, ^13^C, DEPT spectra (Table 1) and UV absorption data of **6** and **4** also allowed identification of these two compounds as analogs. Comparing their spectroscopic data indicated the fatty acyl side chain of **6** has three methylenes more than **4**, which was deduced by the ^1^H, ^13^C, DEPT, HSQC and HMBC spectra of **6**. The ^13^C and ^1^H assignments of the fatty acyl side chain of **6** were achieved by its ^1^H-^1^H COSY, HSQC and HMBC spectra and ACD/Lab 6.0 software. **6** was identified as 23-(9-methyl)undecanoic acid demalonylazalomycin F_4a_ ester.

23-(10-Methyl)undecanoic acid demalonylazalomycin F_4a_ ester (**7**) was obtained as a white, amorphous powder with [α]_D_^20^ +6.0° (*c* 0.1, MeOH). Its molecular formula C_65_H_115_N_3_O_15_ was established by the HRESIMS spectrometric data at *m*/*z* 1178.8426 [M + H]^+^ (calcd. for C_65_H_116_N_3_O_15_, 1178.8406). The difference of 14 mass units between **7** and **6** indicated that **7** has one methylene unit more than **6**. Similar ^1^H, ^13^C, DEPT spectra and UV absorption data of them allowed identification of these two compounds as analogs. Comparing their ^1^H, ^13^C and DEPT spectra, there was no obvious difference between the ^13^C NMR spectrum of **7** and **6** except that one methylene carbon signal at about δ_C_ 31.0 was presented in the ^13^C NMR spectrum of **7**. So, **7** was identified as 23-(10-methyl)decanoic acid demalonylazalomycin F_4a_ ester.

**Figure 4:**
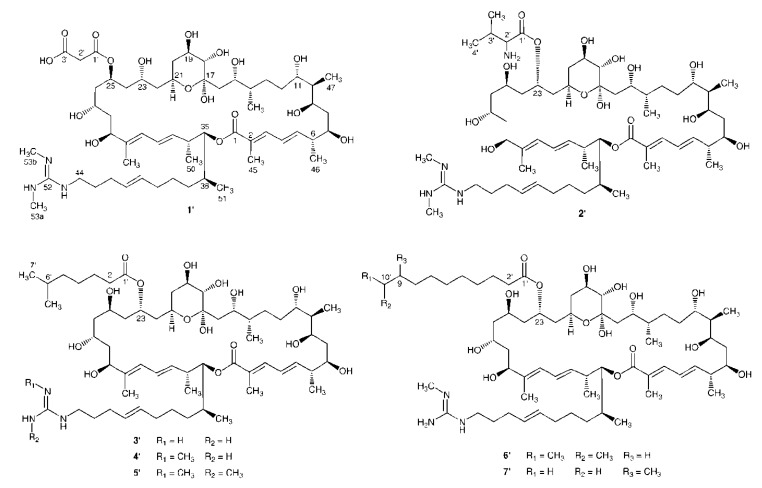
Structures of **1**′–**7**′.

After the planar structures of **1**–**7** were established, we focused on their stereochemistries. As the core macrolide planar structures of **1**–**7** were accordingly identical to those of azalomycins F_5a_, F_4a_ or F_3a_, the relative configurations of the core macrolide structures of **1**–**7** except the structural fragment from C-23 to C-27 of **1** could be directly established by comparing their ^13^C and ^1^H NMR spectra with those of azalomycins F_5a_, F_4a_ or F_3a_ [11,12,13,15,16]. Similar spectroscopic data of their core macrolide structures deduced that the relative configurations of **1**–**7** except that at C-23/C-25/C-27 of **1** were accordingly identical to those of azalomycins F_5a_, F_4a_ or F_3a_. Like that of azalomycin F_5a_, the chemical shifts for C-21 (65.7 ppm), C-23 (66.3 ppm) and C-27 (65.7 ppm) lower than 68.0 ppm in MeOH-*d*_4_ deduced that the relavitve configuration at C-23/C-25/C-27 of **1** was also *anti*/*anti* according to the universal NMR Database **4** [12,17], which was further confirmed by two facts that **1** could be convertible to azalomycin F_5a_ in MeOH-*d*_4_ at room temperature and that the chemical shift for C-23 was upfield by about 5.0 ppm when the malonyl group of azalomycin F_4a_ was removed [12]. Because the relative stereochemistries of C_6_–C_11_ to C_14_–C_36_stereogenic centers of azalomycins F_5a_ remain undefined in our previous work [12], azalomycin F_5a_ was one of two possible stereoisomers which the relative configuration at C_11_/C_14_ was *anti* or *syn*. Similarly, azalomycins F_4a_ and F_3a_ were respectively one of two possible stereoisomers like that of azalomycin F_5a_. So, each compound of **1**–**7** was one of two possible stereoisomers numbered **1**–**7** and **1**′–**7**′ presented in Figure 1, Figure 4, respectively.

### 2.2. Biological Assays

Biological assays indicated that **1**–**7** had broad-spectrum antimicrobial activity. Their minimal inhibitory concentrations (MICs) against *Candida albicans* ATCC 10231, *Staphylococcus aureus* S014, *Bacillus subtilis* S028 and *Escherichia coli* S002 were respectively 1.56–6.25, 0.39–1.56, 0.20–0.78 and 3.13–25.00 μg/mL (Table 2). Moreover, they also showed moderate cytotoxicity against human colon tumor cell HCT-116 *in vitro* with IC_50_ values of 1.81–5.00 μg/mL (Table 2).

**Table 2:** Minimal inhibitory concentrations (MICs) against test microbes and IC_50_ value against HCT-116 *in vitro*.

Compounds	MICs (μg/mL)				IC_50_ (μg/mL)
	*Candida albicans* ATCC 10231	*Staphylococcus aureus* S014	*Bacillus subtilis* S028	*Eschzerichia coli* S002	HCT-116
1	3.13	0.39	0.20	3.13	5.00
2	6.25	1.56	0.39	6.25	1.95
3	3.13	0.78	0.39	3.13	2.46
4	1.56	1.56	0.20	6.25	2.45
5	1.56	0.78	0.78	12.5	1.81
6	3.13	0.39	0.39	25.00	1.54
7	3.13	0.39	0.39	3.13	2.46
Positive controls *	2.0	0.50	0.20	2.0	0.18

* Amphotericin B for *C*. *albicans*, oxacillin sodium for *S*. *aureus* and *B*. *subtilis*, kalamycin for *E*. *coli* and doxorubicin for HCT-116 were respectively used as positive controls.

### 2.3. Discussion

Azalomycin F complex, including azalomycins F_3a_, F_4a_, F_5a_ and several minor analogs, was first isolated from the broth of *Streptomyces hygroscopicus* var. *azalomyceticus* by Arai in 1959 [18,19]. The structures of azalomycins F_3a_, F_4a_ and F_5a_ were progressively elucidated from 1982 to 2012 [12,14,20,21,22], while others minor analogs were not identified. *Streptomyces* sp. 211726, isolated from mangrove rhizosphere soil, showed a remarkable productivity of macrocyclic lactones, and five main components azalomycins F_3a_, F_4a_, F_5a_azalomycin F_4a_ 2-ethylpentyl ester and azalomycin F_5a_ 2-ethylpentyl ester produced by this strain were identified [11]. During our research on minor analogs produced by this strain, seven new compounds **1**–**7** were isolated and identified in this paper. Similar to these azalomycin F analogs, many 36-membered polyhydroxyl macrolides such as RS-22, guanidylfungins, amycins, niphimycin, malonylniphimycin, dihydroniphimycin, malonyl-4,5-dihydroniphimycin, shurimycins and others were identified [23,24,25,26]. There are about thirty 36-membered polyhydroxyl macrolides identified so far, and almost all of them were produced by *Streptomyces*. These compounds possess broad-spectrum antimicrobial activity, and azalomycin F was also an inhibitor of type-I interleukin-1 receptor [27]. In our research on biological activity of azalomycin F analogs produced by *Streptomyces* sp. 211726, these twelve compounds also showed remarkable broad-spectrum antimicrobial activity. Moreover, they had moderate cytotoxicity, and the acute toxicity (LD_50_) of a mixture of twelve azalomycin F analogs produced by this strain was 97.9 mg/kg in mice by intraperitoneal injection.

## 3. Experimental Section

### 3.1. General Experimental Procedures

Optical rotations were measured in methanol using an Autopol Ш digital polarimeter. UV spectra were determined by DU-800 UV/Visible spectrophotometer. IR spectra were obtained with Thermo Nicolet 380 FTIR spectrometer. All NMR experiments were recorded on a Bruker AV-400 NMR spectrometer equipped with a 5 mm PABBO BB-probe head (400 MHz for ^1^H shifts relative to MeOH-*d*_4_ at 3.31 ppm and 100 MHz for ^13^C shifts relative to MeOH-*d*_4_ at 49.05 ppm) at 30 °C. HR-ESI-MS spectra were carried on the API QSTAR Pulsar I MS System. Silica gel (Qingdao Haiyang Chemical Co. Ltd., Qingdao, China, 10–40 μm), octadecylsilyl silica gel (Silicycle, Quebec, Canada; 40–63 μm) and Sephadex LH-20 (Amersham Pharmacia Biotech AB, Uppsala, Sweden) were used for column chromatography. Precoated silica gel GF_254_ plates (Qingdao Haiyang Chemical Co., Ltd.) were used for thin layer chromatography. All compounds was prepared by Dionex Summit HPLC system (Dionex, Sunnyvale, CA, USA) consisting of Dionex P680 HPLC pumps (P680A HPG-4) with a UV detector (170 U), and a Shimadzu Shim-pack VP-ODS reversed-phase column (250 mm × 4.6 mm i.d., 5-μm particle size) was used.

### 3.2. Actinomycetes Material and Fermentation

The strain of *Streptomyces* sp. 211726 was isolated from mangrove rhizosphere soil of *Heritiera globosa* collected in Wenchang, China. Voucher specimens are stored in Key Laboratory of Combinatorial Biosynthesis and Drug Discovery (Wuhan University), Ministry of Education, Wuhan, China. The fermentation of strain*Streptomyces* sp. 211726 was reported in our previous paper [11]. In short, the strain of *Streptomyces* sp. 211726 was cultured with liquid medium containing glucose 1.0%, soluble starch 3.5%, yeast 0.2%, casein 0.4% and NaCl 1.8% (pH 7.2 before autoclaving), and incubated at 30 °C for 10 days on a rotary shaker at 190 rpm until 100 L of broth was obtained.

### 3.3. Extract and Isolation

After the 100 L broth of *Streptomyces* sp. 211726 was centrifuged, the mycelia was extracted with methanol, concentrated under vacuum and freeze-dried to obtain lyophilized powder. The powder was dissolve in CHCl_3_:MeOH (80:20), and separated into eight fractions (1–8) on a silica gel column using gradient elution of CHCl_3_:MeOH (3:1, 2:1 and 1:1). Fraction 2 was purified by reversed-phase C_18_ column eluted with MeOH:H_2_O (60:40), semi-preparative reversed-phase C_18_ high performance liquid chromatography eluted with MeOH:H_2_O (58:42) to give three pure fraction, and which were respectively concentrated under vacuum to obtain three extracts. These extracts were respectively dissolved in MeOH, purified by sephadex LH-20 column eluted with MeOH, concentrated under vacuum at 35 °C, and dried to give **1** (41 mg), **2** (22 mg) and **3** (31 mg). Similarly, fraction 3 was purified by reversed-phase C_18_ column eluted with MeOH:H_2_O (65:35), semi-preparative reversed-phase C_18_ high performance liquid chromatography eluted with MeOH:H_2_O (63:37) and sephadex LH-20 column eluted with MeOH to give **4** (15 mg) and **6** (27 mg); Fraction 4 was purified by reversed-phase C_18_ column eluted with MeOH:H_2_O (70:30), semi-preparative reversed-phase C_18_ high performance liquid chromatography eluted with MeOH:H_2_O (68:32) and sephadex LH-20 column eluted with MeOH to give **5** (10 mg) and **7** (24 mg).

25-Malonyl demalonylazalomycin F_5a_ monoester (**1**): White amorphous powder; [α]_D_^29^ +6.7° (*c* 0.1, MeOH); UV λ^MeOH^_max_ nm (log ε): 241(4.6), 269(4.3); IR υ^KBr^_max_ (cm^−1^): 3385, 2964, 2936, 1701, 1636, 1597, 1459, 1243, 1089, 1066, 969; ^13^C NMR (MeOH-*d*_4_, 100 MHz) and ^1^H NMR (MeOH-*d*_4_, 400 MHz) data were showed in Table 1; HRESIMS *m*/*z* 1096.6914 [M + H]^+^ (calcd. for C_57_H_98_N_3_O_17_, 1096.6896).

23-Valine demalonylazalomycin F_5a_ ester (**2**): White amorphous powder; [α]_D_^29^ +4.4° (*c* 0.1, MeOH); UV λ^MeOH^_max_ nm (log ε): 241(4.6), 268(4.4); IR υ^KBr^_max_ (cm^−1^): 3414, 3137, 2965, 2928, 1726, 1635, 1597, 1507, 1261, 1092, 968; ^13^C NMR (MeOH-*d*_4_, 100 MHz) and ^1^H NMR (MeOH-*d*_4_, 400 MHz) data were showed in Table 1; HRESIMS *m*/*z* 1109.7580 [M + H]^+^ (calcd. for C_59_H_105_N_4_O_15_, 1109.7576).

23-(6-Methyl)heptanoic acid demalonylazalomycin F_3a_ ester (**3**): White amorphous powder; [α]_D_^20^ +6.8° (*c* 0.1, MeOH); UV λ^MeOH^_max_ nm (log ε): 238(4.6), 269(4.3); IR υ^KBr^_max_ (cm^−1^): 3423, 2962, 2935, 1736, 1707, 1637, 1184, 1045, 970, 721; ^13^C NMR (MeOH-*d*_4_, 100 MHz) and ^1^H NMR (MeOH-*d*_4_, 400 MHz) data were showed in Table 1; HRESIMS *m*/*z* 1108.7638 [M + H]^+^ (calcd. for C_60_H_106_N_3_O_15_, 1108.7624).

23-(6-Methyl)heptanoic acid demalonylazalomycin F_4a_ ester (**4**): White amorphous powder; [α]_D_^20^ +6.4° (*c* 0.1, MeOH); UV λ^MeOH^_max_ nm (log ε): 238(4.5), 269(4.4); IR υ^KBr^_max_ (cm^−1^): 3423, 2965, 2936, 1734, 1708, 1635, 1181, 1049, 972, 722; ^1^H NMR (MeOH-*d*_4_, 400 MHz) and ^13^C NMR (MeOH-*d*_4_, 100 MHz) data were showed in supplementary file; HRESIMS *m*/*z* 1122.7788 [M + H]^+^ (calcd. for C_61_H_108_N_3_O_15_, 1122.7780).

23-(6-Methyl)heptanoic acid demalonylazalomycin F_5a_ ester (**5)**: White amorphous powder; [α]_D_^20^ +6.1° (*c* 0.1, MeOH); UV λ^MeOH^_max_ nm (log ε): 238(4.5), 269(4.4); IR υ^KBr^_max_ (cm^−1^): 3425, 2963, 2935, 1734, 1708, 1636, 1185, 1047, 972, 721; ^1^H NMR (MeOH-*d*_4_, 400 MHz) and ^13^C NMR (MeOH-*d*_4_, 100 MHz) data were showed in supplementary file; HRESIMS *m*/*z* 1136.7956 [M + H]^+^ (calcd. for C_62_H_110_N_3_O_15_, 1136.7937).

23-(9-Methyl)decanoic acid demalonylazalomycin F_4a_ ester (**6**): White amorphous powder; [α]_D_^20^ +6.0° (*c* 0.1, MeOH); UV λ^MeOH^_max_ nm (log ε): 238(4.5), 269(4.4); IR υ^KBr^_max_ (cm^−1^): 3425, 2968, 2935, 1734, 1708, 1636, 1185, 1047, 972, 721; ^1^H NMR (MeOH-*d*_4_, 400 MHz) and ^13^C NMR (MeOH-*d*_4_, 100 MHz) data were showed in Table 1; HRESIMS *m*/*z* 1164.8269 [M + H]^+^ (calcd. for C_64_H_114_N_3_O_15_, 1164.8250).

23-(10-Methyl)undecanoic acid demalonylazalomycin F_4a_ ester (**7**): White amorphous powder; [α]_D_^20^ +6.0° (*c* 0.1, MeOH); UV λ^MeOH^_max_ nm (log ε): 238(4.5), 269(4.4); IR υ (cm^−1^): 3425, 2968, 2936, 1736, 1707, 1636, 1185, 1047, 972, 721; ^13^C NMR (MeOH-*d*_4_, 100 MHz) and ^1^H NMR (MeOH-*d*_4_, 400 MHz) data were showed in supplementary file; HRESIMS *m*/*z* 1178.8426 [M + H]^+^ (calcd. for C_65_H_116_N_3_O_15_, 1178.8406).

### 3.4. Biological Assays

The MICs of all compounds against *C*. *albicans* ATCC 10231, *S*. *aureus* S014, *B*. *subtilis* S028 and *E*. *coli* S002 were determined by agar dilution method. Amphotericin B for *C*. *albicans*, oxacillin sodiumfor *S*. *aureus* and *B*. *subtilis* and kalamycin for *E*. *coli* were respectively used as positive controls. Yeast extract-peptone-dextrose (YPD) medium was used for *C. albicans*, beef extract-peptone medium was used for *S. aureus* and *B. subtilis*, and Luria-Bertani (LB) medium was used for *E*. *coli*. Their cytotoxicities were evaluated by the MTT (3-(4,5-dimethylthiazol-2-yl)-2,5-diphenyl-tetrazolium bromide) method using human colon tumor cell HCT-116, and doxorubicin was used as a positive control.

## 4. Conclusions

Proceed with research on minor azalomycin F analogs produced by *Streptomyces* sp. 211726, seven new compounds were isolated and identified. Biological assays of **1**–**7** showed remarkable antifungal and antibacterial activity and moderate cytotoxicity against human colon tumor cell HCT-116 *in vitro*.

## Supplementary Files

Supplementary File 1 Supplementary Information (PDF, 432 KB)
