# Supplementary material for: New Azalomycin F Analogs from Mangrove Streptomyces sp. 211726 with Activity against Microbes and Cancer Cells
Source: Mar Drugs. 2013 Mar 12;11(3):817–29. doi: 10.3390/md11030817 (PMC3705372; doi:10.3390/md11030817)
Supplement: Supplementary File 1 — Supplementary Information (PDF, 432 KB) [file marinedrugs-11-00817-s001.pdf]

Supplementary Information

Table S1. NMR spectroscopic data (400 MHz for ^{1}H, 100 MHz for ^{13}C) of **4**, **5** and **7** in MeOH-d_4 (δ in ppm).

Position	**4**		**5**		**7**	
	δ_C	δ_H (J in Hz)	δ_C	δ_H (J in Hz)	δ_C	δ_H (J in Hz)
C-1	170.1	-	170.1	-	170.1	-
C-2	126.8	-	126.7	-	126.8	-
C-3	140.2	7.10 d (11.2)	140.2	7.09 d (11.2)	140.3	7.10 d (11.0)
C-4	127.6	6.43 dd (11.9, 14.4)	127.6	6.45 dd (11.9, 14.4)	127.6	6.43 dd (11.5, 14.9)
C-5	146.1	6.08 dd (14.0, 9.5)	146.1	6.08 dd (14.0, 9.6)	146.2	6.08 dd (14.9, 9.0)
C-6	44.5	2.44 m	44.5	2.43 m	44.7	2.44 m
C-7	75.8	3.77 m	75.8	3.76 m	75.8	3.77 m
C-8	39.3	1.50 m, 1.77 m	39.3	1.48 m, 1.78 m	39.3	1.50 m, 1.78 m
C-9	75.3	3.80 m	75.3	3.78 m	75.2	3.80 m
C-10	44.6	1.54 m	44.6	1.52 m	44.6	1.53 m
C-11	72.2	3.91 m	72.3	3.92 m	72.2	3.87 m
C-12	33.4	1.62 m, 1.75 m	33.5	1.61 m, 1.75 m	33.4	1.60 m, 1.36 m
C-13	30.6	1.30 m, 1.43 m	30.7	1.28 m, 1.45 m	30.6	1.30 m, 1.44 m
C-14	40.5	1.61 m	40.5	1.60 m	40.5	1.61 m
C-15	72.4	3.86 m	72.4	3.85 m	72.7	3.87 m
C-16	41.9	1.82 m	42.0	1.82 m	42.0	1.81 m
C-17	99.9	-	100.0	-	99.9	-
C-18	77.5	3.35 d (9.2)	77.3	3.35 d (9.2)	77.5	3.35 d (9.1)
C-19	69.8	3.87 m	69.7	3.87 m	69.8	3.88 m
C-20	41.3	1.90 m, 1.31 m	41.4	1.90 m, 1.30 m	41.3	1.89 m, 1.30 m
C-21	66.3	4.16 m	66.4	4.15 m	66.3	4.16 m
C-22	41.9	1.85 m	41.9	1.86 m	41.9	1.85 m
C-23	70.9	5.27 m	70.8	5.25 m	70.9	5.29 m
C-24	44.1	1.72 m	44.1	1.72 m	44.0	1.70 m, 1.63 m
C-25	65.8	3.86 m	65.7	3.87 m	65.8	3.86 m
C-26	46.4	1.50 m	46.4	1.49 m	46.3	1.51 m
C-27	65.7	4.04 m	65.6	4.02 m	65.7	4.04 m
C-28	44.1	1.54 m	44.1	1.53 m	44.1	1.64 m
C-29	74.8	4.18 m	74.1	4.17 m	74.2	4.18 m
C-30	140.1	-	140.1	-	140.1	-
C-31	125.3	5.98 d (10.6)	125.2	5.98 d (10.6)	125.3	5.98 d (10.7)
C-32	128.5	6.22 dd (10.9, 14.8)	128.5	6.21 dd (10.9, 14.8)	128.5	6.23 dd (10.9, 14.8)
C-33	136.3	5.45 m	136.3	5.42 m	136.3	5.44 m
C-34	40.9	2.57 m	40.9	2.56 m	40.7	2.57 m
C-35	80.9	4.78 dd (7.6, 4.0)	80.9	4.77 dd (7.8, 3.9)	80.9	4.79 dd (7.6, 4.1)
C-36	35.3	1.81 m	35.2	1.81 m	35.3	1.82 m
C-37	34.3	1.15 m, 1.35 m	34.3	1.15, 1.33 m	34.4	1.15 m, 1.35 m
C-38	27.9	1.41 m	27.9	1.40 m	27.9	1.42 m
C-39	33.6	1.99 m	33.6	1.98 m	33.6	1.99 m
C-40	132.6	5.44 m	132.5	5.49 m	132.7	5.44 m
C-41	130.3	5.50 m	130.3	5.43 m	130.2	5.44 m

Table S1. *Cont.*

C-42	30.8	2.07 m	30.7	2.06 m	30.6	2.07 m
C-43	29.9	1.64 m	29.8	1.64 m	29.9	1.64 m
C-44	42.0	3.15 t (7.0)	42.0	3.15 t (7.0)	42.0	3.15 t (7.1)
C-45	12.9	1.92 s	12.9	1.90 s	12.9	1.92 s
C-46	17.2	1.11 d (6.8)	17.1	1.10 d (6.8)	17.1	1.12 d (6.8)
C-47	10.5	0.89 d (6.9)	10.5	0.87 d (6.9)	10.5	0.89 d (6.9)
C-48	15.3	0.91 d (6.7)	14.9	0.90 d (6.7)	15.3	0.92 d (6.7)
C-49	13.1	1.65 s	13.3	1.63 s	13.1	1.65 s
C-50	17.8	1.00 d (6.6)	17.9	1.00 d (6.6)	17.8	1.00 d (6.8)
C-51	14.5	0.94 d (6.7)	14.3	0.93 d (6.7)	14.5	0.94 d (6.7)
C-52	158.3	-	157.4	-	158.3	-
C-53a	28.4	2.84 s	28.4	2.84 s	28.4	2.84 s
C-53b			28.4	2.84 s		
C-1′	175.4	-	175.4	-	175.5	-
C-2′	35.0	2.36 t (7.4)	35.0	2.35 t (7.4)	34.9	2.36 t (7.5)
C-3′	26.0	1.62 m	26.0	1.61 m	26.0	1.61 m
C-4′	30.5	1.42 m	30.5	1.42 m	30.3	1.35 m
C-5′	40.3	1.18 m	40.3	1.17 m	30.5	1.31 m
C-6′	29.2	1.29 m	29.2	1.29 m	30.8	1.30 m
6′-CH_3	23.1	0.88 d (6.6)	23.1	0.87 d (6.6)		
C-7′	23.1	0.88 d (6.6)	23.1	0.87 d (6.6)	31.0	1.30 m
C-8′					28.5	1.29 m
C-9′					40.3	1.17 m
C-10′					29.2	1.31 m
10′-CH_3					23.1	0.89 d (6.8)
C-11′					23.1	0.89 d (6.8)

Figure S1. ^{13}C NMR spectum (100 MHz) of **1** in MeOH-d_4.

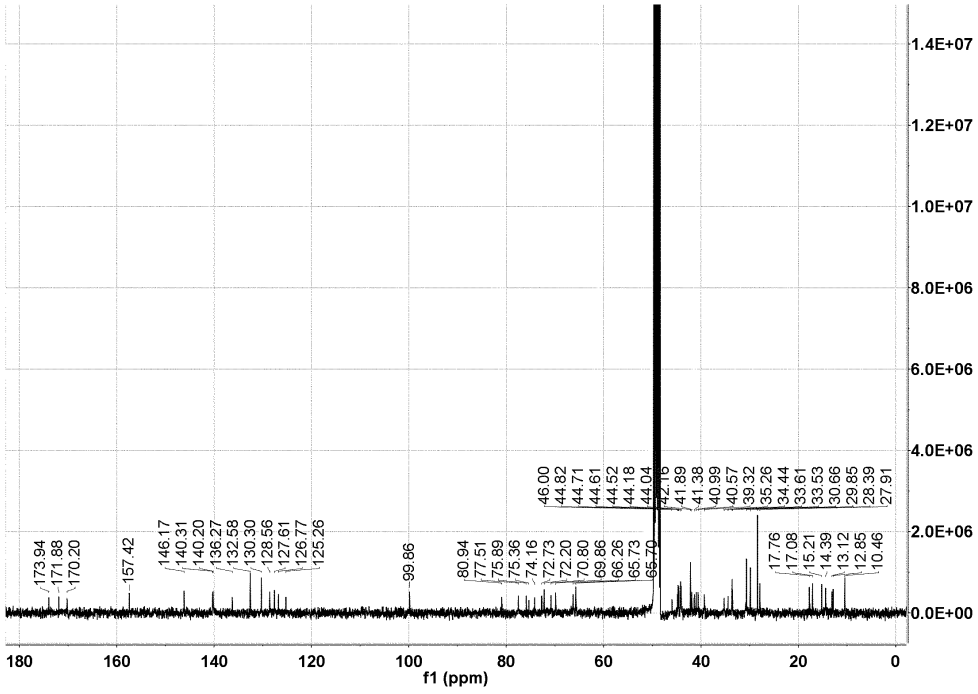

Figure S2. ^{1}H NMR spectum (400 MHz) of **1** in MeOH-d_4.

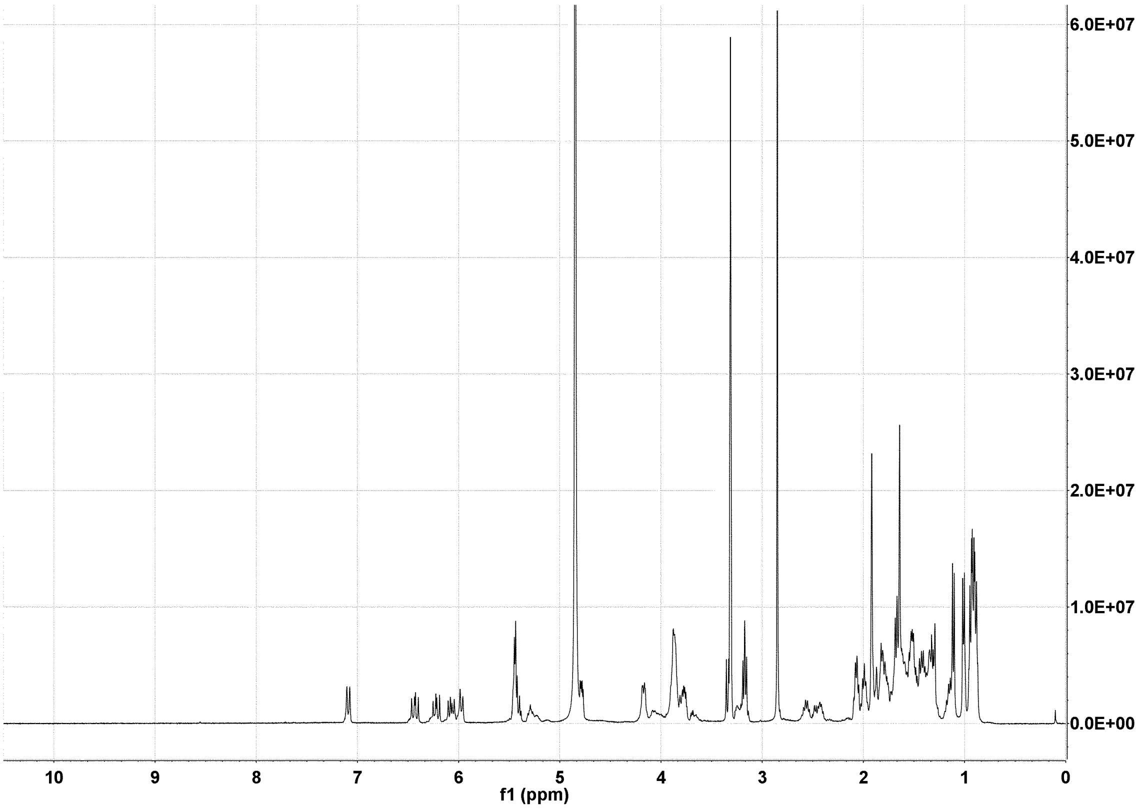

Figure S3. ^{13}C NMR spectum (100 MHz) of **2** in MeOH-d_4.

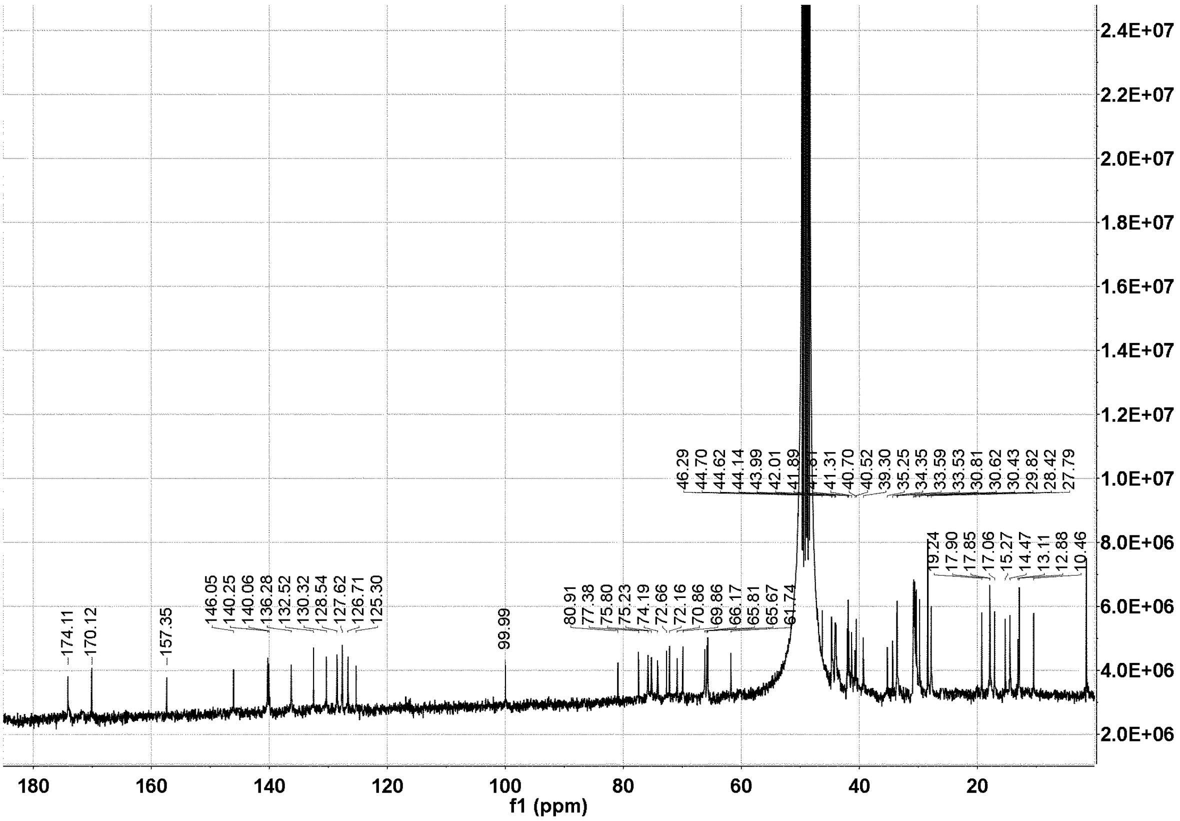

Figure S4. ^{1}H NMR spectum (400 MHz) of **2** in MeOH-d_4.

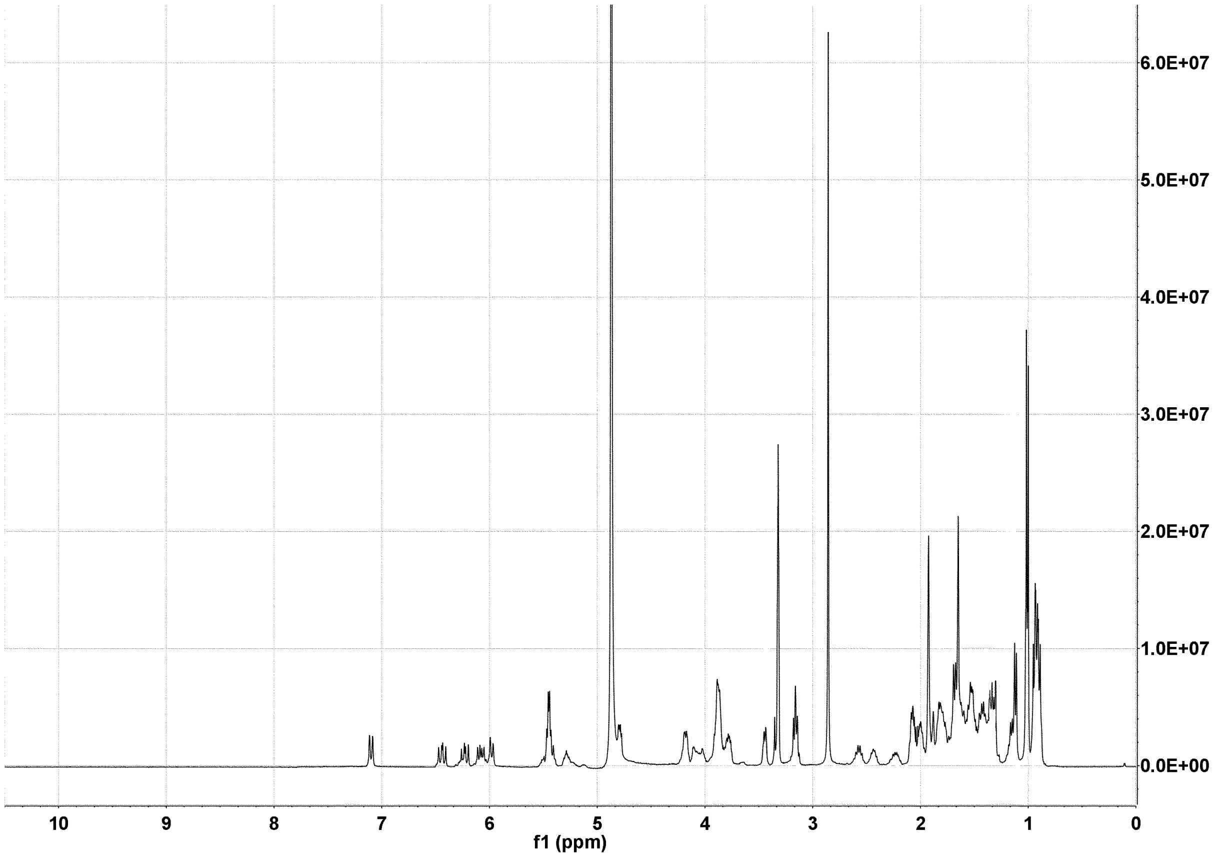

Figure S5. ^{13}C NMR spectum (100 MHz) of **3** in MeOH-d_4.

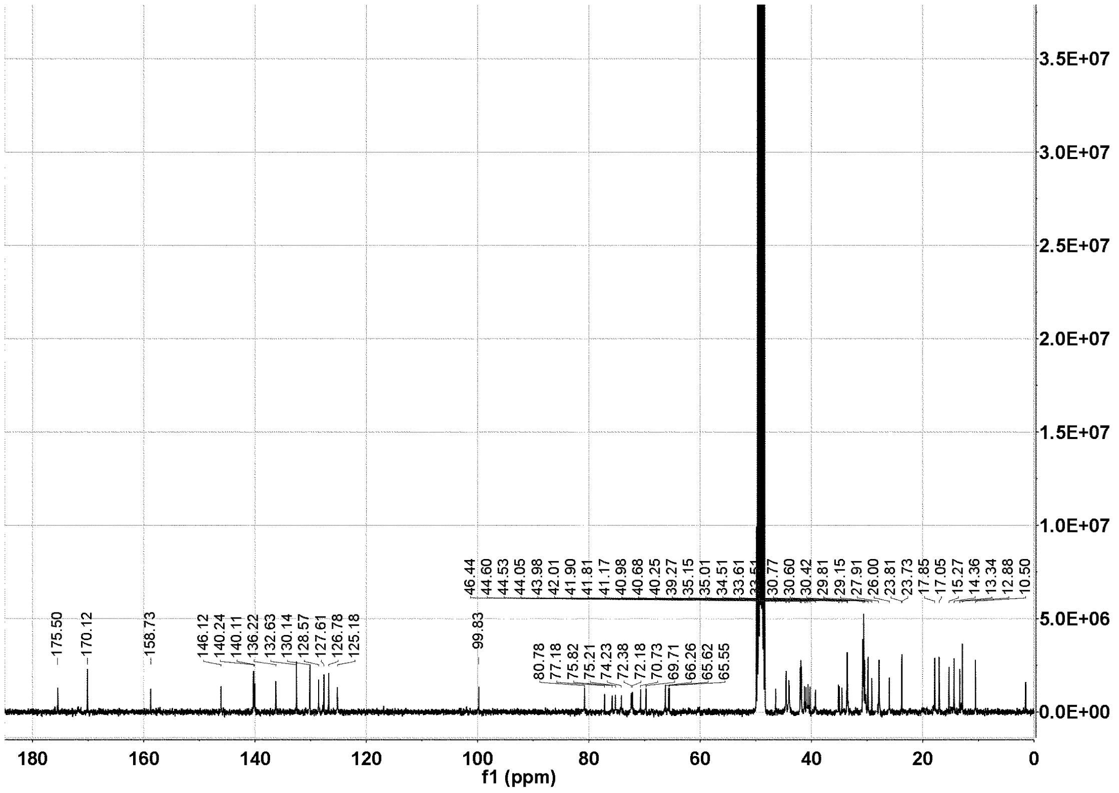

Figure S6. ^{1}H NMR spectum (400 MHz) of **3** in MeOH-d_4.

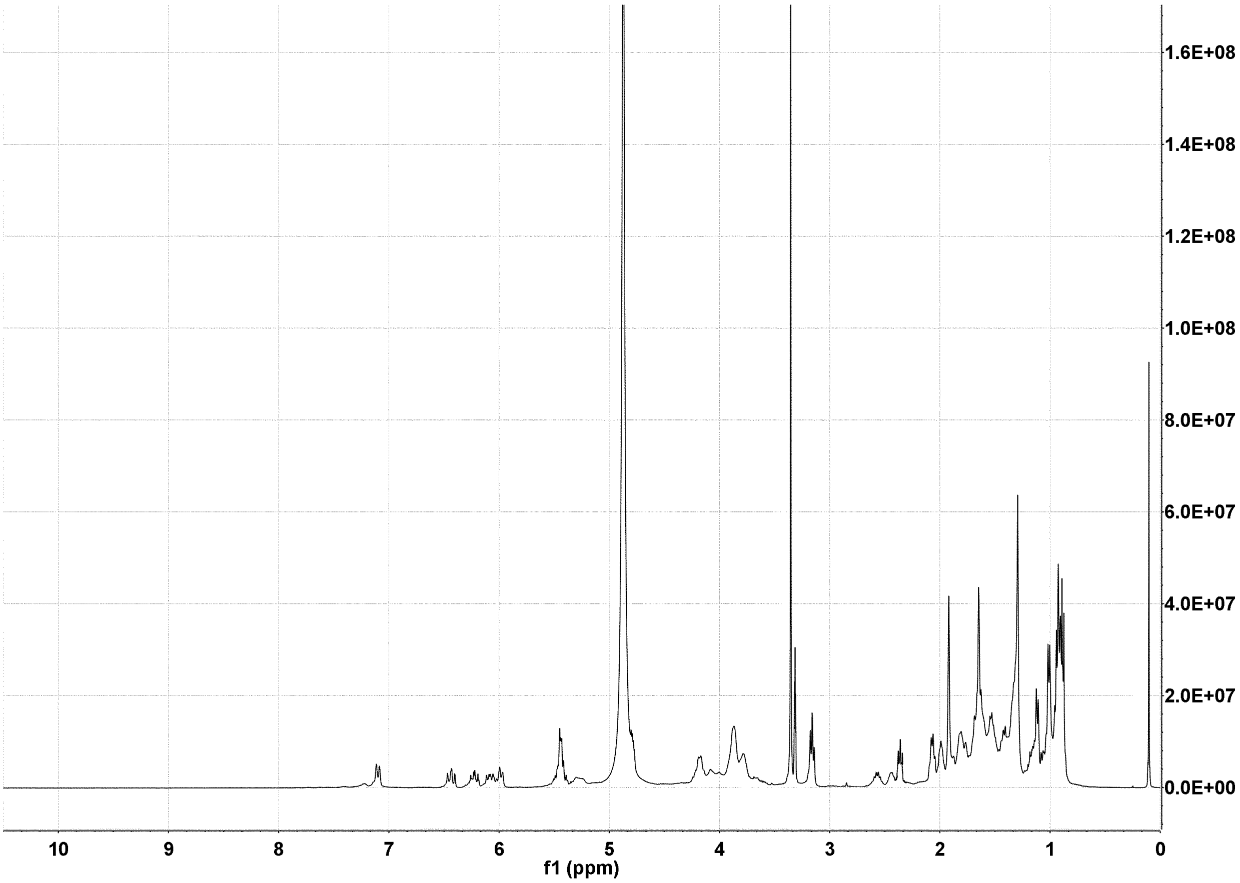

Figure S7. ^{13}C NMR spectum (100 MHz) of **6** in MeOH-d_4.

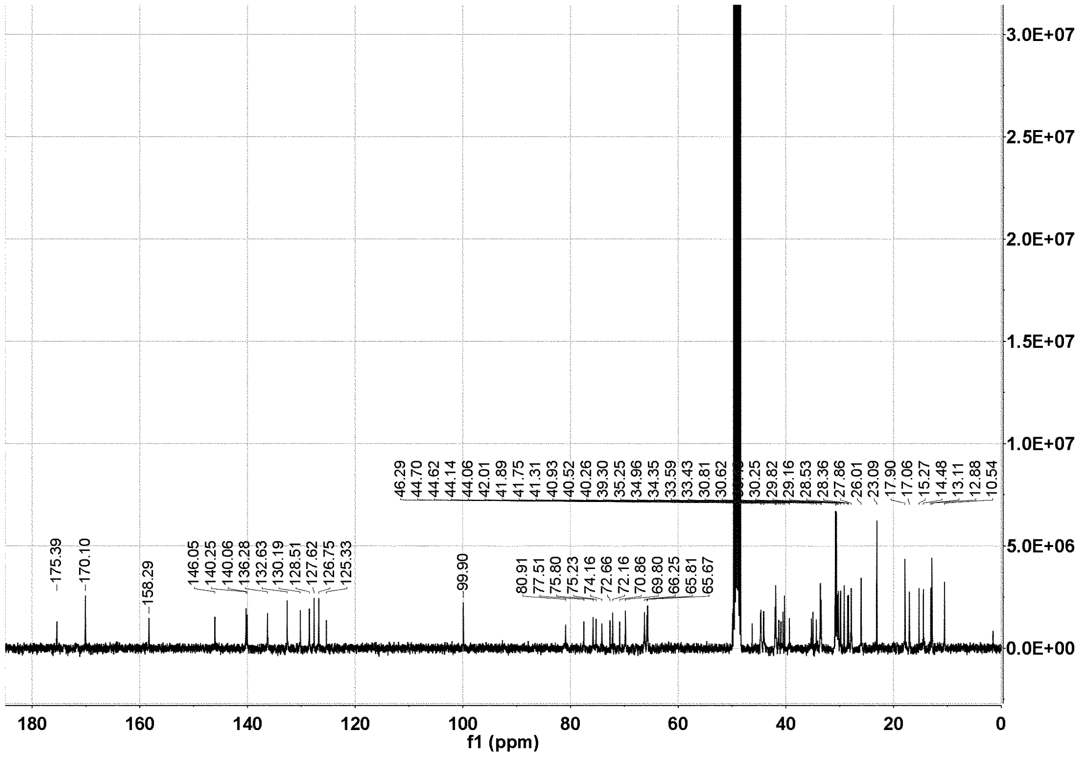

Figure S8. ^{1}H NMR spectum (400 MHz) of **6** in MeOH-d_4.

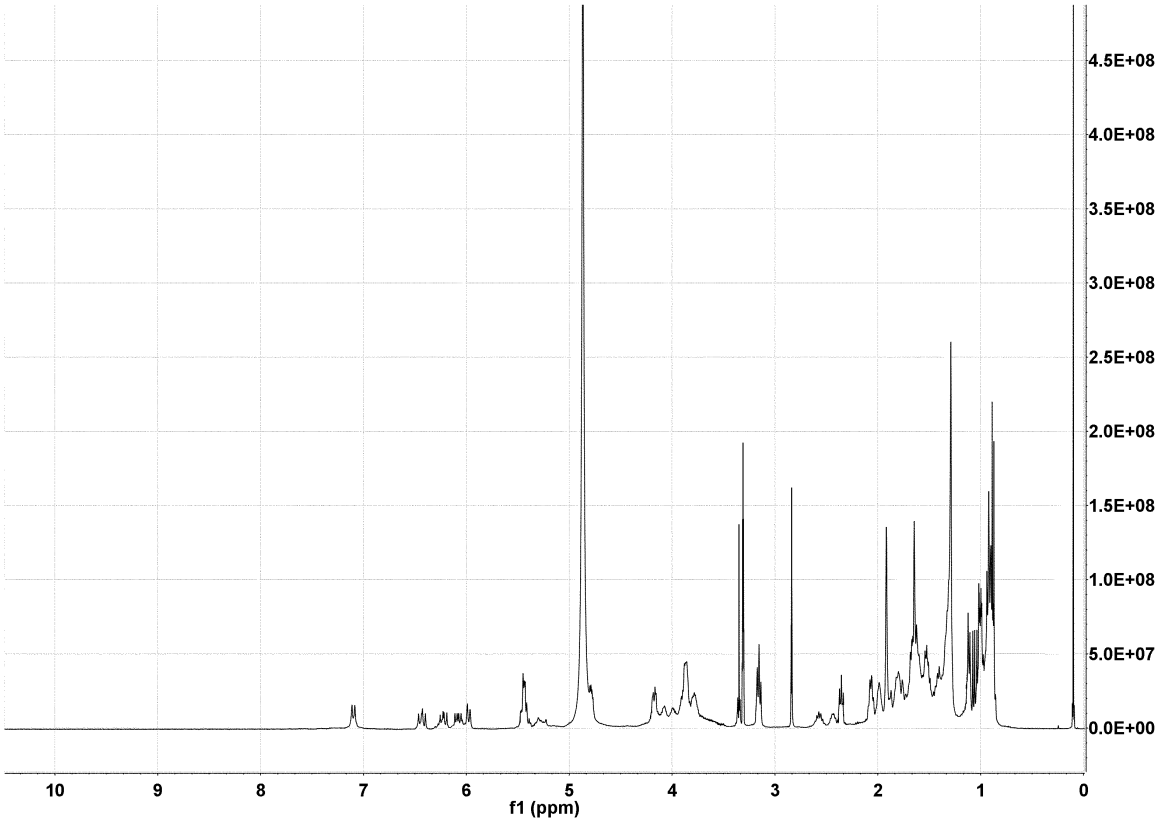

© 2013 by the authors; licensee MDPI, Basel, Switzerland. This article is an open access article distributed under the terms and conditions of the Creative Commons Attribution license (http://creativecommons.org/licenses/by/3.0/).
